# Personal and sexual boundaries: the experiences of people with intellectual disabilities

**DOI:** 10.1186/s12889-022-14181-x

**Published:** 2022-09-19

**Authors:** Gøril Brevik Svae, Line Blixt, Erik Søndenaa

**Affiliations:** 1 Department of Neurohabilitation/Oslo University Hospital, Kirkeveien 166, 0450 Oslo, Norway; 2 Division of Clinical Neuroscience. Department of Research and Innovation, Oslo University Hospital, Oslo, Norway; 3 Institute of Clinical Medicine, University of Oslo, Oslo, Norway; 4 Institute for Health, Faculty of Health Studies, Institute of Health, VID Specialized University, Stavanger, Norway; 5 Department of Mental Health, Norwegian University of Science and Technology, Trondheim, Norway; 6 Centre for Research & Education in Forensic Psychiatry, St. Olav’s Hospital, Trondheim, Norway

**Keywords:** Intellectual disability, Sexual consent, Personal and sexual boundaries, Sexual abuse, Trauma, Police investigation

## Abstract

**Background:**

Previous research shows that people with intellectual disabilities have less knowledge about sexual health and are more vulnerable to victimisation. In cases of sexual abuse, they are likely to encounter the criminal justice system as vulnerable witnesses. Several challenges arise when people with intellectual disabilities are in communication with the criminal justice system. We aimed to explore the perceptions, experiences and knowledge of people with intellectual disabilities regarding personal and sexual boundaries in order to identify factors relevant for preventing sexual abuse, to develop future studies.

**Method:**

The study had a qualitative design. Data were collected from seven people with mild intellectual disabilities (25–40 years; 2 men, five women) through one-to-one interviews in specialised health care services for people with intellectual disabilities (SHCS). The participants lived alone, in group homes and with their families. Many received professional support services. Data were analysed using thematic analysis.

**Results:**

The interviews identified that the participants want to be in romantic relationships and that some, consider sex to be unimportant. Many of them have had trouble finding someone to have a romantic relationship with. The participants were unsure about sexual consent related to sexual activity, though many could explain the concept of consent in other contexts. Many participants reported that they had experienced sexual abuse, including online sexual abuse. Those participants who had reported the sexual abuse had positive experiences obtaining assistance from the criminal justice system. The participants who had experienced sexual abuse reported trauma and fear related to their experiences.

**Conclusion:**

This study highlights the need for information about sexually abusive relationships, risks online and ways to get help. More attention should be given to the impact of trauma, police and mental health treatment following sexual abuse against people with intellectual disabilities.

## Background

Sexual health is a fundamental aspect of life for human beings [1], and people with intellectual disabilities have voiced that sexuality is an essential topic [2]. However, there are several barriers to ensuring that people with intellectual disabilities have good sexual health. The World Health Organisation (WHO) has stated that the sexual health and well-being depends on individual knowledge of potential risks and their vulnerability to the negative consequences of risky sexual activity [3]. We know that people with intellectual disabilities have less knowledge about sexual health and less sexual experience compared with people without disabilities [4–7]. Further, people with intellectual disabilities have little knowledge about laws related to sexual relationships, such as the age of consent and what constitutes abuse [8]. This lack of information can make it more challenging for people with intellectual disabilities to navigate the sexual landscape, leaving them in unsafe situations and at an increased risk of abuse [9]. Adults with intellectual disabilities want meaningful relationships and intimacy [10]. Research has shown that many with intellectual disabilities desire a romantic relationship and hold a positive view of being in a such relation [11, 12]. A study by Lesseliers and Van Hove [13] has shown that people with intellectual disabilities do not wish to have sexual intercourse but are satisfied with kissing and cuddling in a relationship. A study by Eastgate, van Driel [14] found that people with intellectual disabilities struggle to decline sex they did not want to have within a relationship. Sullivan, Bowden [15] found that people with intellectual disabilities held negative perceptions of sexual behaviour within relations. Comprised, these studies may indicate that people with intellectual disabilities have little sexual knowledge regarding sexual activity and feel insecure about it. Furthermore, some of them may be at risk of abuse and exploitation. Services need to offer protection to prevent harm [16].

One solution suggested by Brown and McCann [17] is better access to education which improves knowledge and the capacity for decision-making regarding relationships. They highlighted the need for more sexual education interventions for people with intellectual disabilities [17]. Several studies have shown that education which provides sexual knowledge can increase the decision-making and protection skills of people with intellectual disabilities [18–20]. The United Nations [21] has stated that people with intellectual disabilities have the same rights to health care and support related to their sexual health as any other human beings. Still, there is a lack of policy guidelines and funding for the support services for people with intellectual disabilities [22].

Research has shown elevated rates of lifetime victimisation among people with intellectual disabilities compared to people without this diagnosis [23]. It has been found that one in three adults with intellectual disabilities has experienced sexual abuse, and that their abusers were often people with intellectual disabilities as well [24]. In a study of 100 participants with intellectual disabilities, Brkić-Jovanović, Runjo [25] found that 82% were sexually active and that most of them had had sex they did not want to due to reduced assertiveness skills. In instances of sexual abuse, it is crucial that the police are aware of any intellectual disabilities in the early stages of a criminal case because this can affect the verdict [26]. Poor communication and a lack of social skills are well-known vulnerabilities among people with an intellectual disability diagnosis [27]. The police frequently meet people they believe to have intellectual disabilities, and communicating with them is a common obstacle [28]. However, good communication is vital since the police are dependent on consistent testimony in court to establish whether a criminal act has been committed [29]. A study by Beckene, Forrester‐Jones [30] found that people with intellectual disabilities may experience court attendance as a further trauma after an incident of abuse due to a lack of support and little understanding of their diagnosis.

As vulnerable witnesses, children and adults with intellectual disabilities in Norway are entitled to a facilitated interview in a Barnahus, a part of the criminal justice sector [31]. One recent study found that 9% of the adults attending a Norwegian sexual assault centre had intellectual disabilities. Of this group, 61% reported that their assailants were family members or acquaintances [32]. Vik, Rasmussen [33] found that these victims were given a low-quality police investigation compared with the other victims. Another Norwegian study looking at cases investigated by the police found that 71.2% of victims with intellectual disabilities were females and that 30% of these cases led to a penal sanction [34]. A study by Søndenaa, Olsen [35] concluded that intellectual disability was detected twice as often in suspects compared with victims in the criminal justice system. The study results showed that all professional parties (police, state attorneys and jurors) reported more frequent contact with suspects with intellectual disabilities than with victims or witnesses with intellectual disabilities. These results may be explained by the fact that the criminal justice system has more contact with suspects of sexual assault with intellectual disabilities, than victims with intellectual disabilities [35].

This study aimed to explore the perceptions and knowledge of adults with intellectual disabilities regarding personal and sexual boundaries. From these insights and experiences, which factors can we identify that future research may consider as potentially relevant to the prevention of sexual abuse?

## Method

A qualitative approach was chosen for this study. Data were collected from semi-structured interviews with seven participants. Data were analysed using thematic analysis in line with Braun and Clarke [36].

### Setting

The participants were recruited from the specialised health care services for people with intellectual disabilities (SHCS) in Oslo, Norway. The SHCS offers counselling and treatment to individuals with intellectual disabilities and their support services in the community. The SHCS supports “difficulties related to sexuality” for people with intellectual disabilities. Treatment can be given for individual conditions like loss of function that affects quality of life, past trauma, the risk of abuse, display of uncritical sexual behaviour, or comorbidity difficulties related to sexuality or pregnancy [37]. The interviews were conducted at the SHCS services in Oslo.

### Ethical considerations

The Regional Committees for Medical and Health Research Ethics of Norway approved the study, approval number 2018/2296. The selection criteria approved by the Regional Committees for Medical and Health Research Ethics required that participants were adults with mild intellectual disabilities receiving specialist SHCS services. We wanted to include people with intellectual disabilities with high function who were being able to talk about their understanding and knowledge of sexual health. The participants were offered support during and following participation in the interviews. According to Norwegian legislation Sect. 196 [38], the research group had a duty to report a criminal offence if a participant disclosed during the interview that they were exposed to ongoing abuse. None of the participants stated that they were being exposed to ongoing assault or abuse at the time of the interview.

### Participants

Clinical specialists as psychologist, nurse and social educator at the SHCS invited service users to participate in the study. A total of seven people (five women and two men) participated (See Table 1).

**Table 1:** A brief description of the participants

P	Sex	Age range	Living condition
1	F	25–30	With family
2	F	35–40	Alone with support
3	M	25–30	Group home
4	M	25–30	Group home
5	F	35–40	Group home
6	F	30–35	Alone without support
7	F	30–35	Alone with support

One person declined from participating in the study. Ages ranged from 25 to 40 years. All participants lived in the community; three lived alone, three in group homes and one lived with the family. Five received professional support. All the participants were employed at the time of the interview. Full confidentiality was guaranteed to the participants who joined the study. Numbers were used in the transcripts to maintain anonymity.

### Data collection

All participants recruited for the study were prepared to talk about sexuality, sexual consent and boundaries related to themselves or others. This briefing was first performed face to face by the clinical specialist who recruited them and repeated by the first author at the beginning of the interview. We made arrangements to ensure that the participants were taken care of and explained to them what was meant by confidentiality. In dialogue with the potential service users, the first author or the clinical specialists at the SHCS offered to talk with someone the participant trusted, such as a family member, or staff from their group home about the study, who could help them understand what participation in the investigation entailed. Information about the study was provided and all participants gave their written informed consent. All the participants were reminded that the interview was voluntary and that they could withdraw from the study at any time. The first author and three experienced clinical specialists from the SHCS in Oslo developed a semi-structured interview guide. During the interviews, the guide’s questions were used as a help to the conversation. This guide allowed for participants to talk more generally about what they knew about sexuality and not necessarily about their own sexual experiences if they did not want to. The first author conducted one-to-one interviews with the participants. She has worked as a clinician for almost 20 years and has extensive experience talking about sexual health with people with intellectual disabilities.

At the start of the interviews, age and sex were registered and all the participants were asked introductory questions like *“Have you had sex education at school*?” and “*Are you in a relationship or do you want a girlfriend/boyfriend*?” Then they moved on to more specific questions about sexuality such as *“What is the difference between a girlfriend/boyfriend and a friend for you?”* and *“What is sexuality?”* The participants were presented with short vignettes with questions about sexual consent and asked for their thoughts about the situations. Questions such as *“Is it ok okay to change your mind about having sex when two people are naked?*” and *“What can you do if you don’t want to have sex”* Later in the interview, we asked: “*Have you had any negative experiences and have you received professional support for your experiences?”.*

The interviews lasted from 15 to 60 min, with an average duration of 35 min. The interviews were audio-recorded and transcribed verbatim by the first author.

### Analysis

**Fig. 1:**
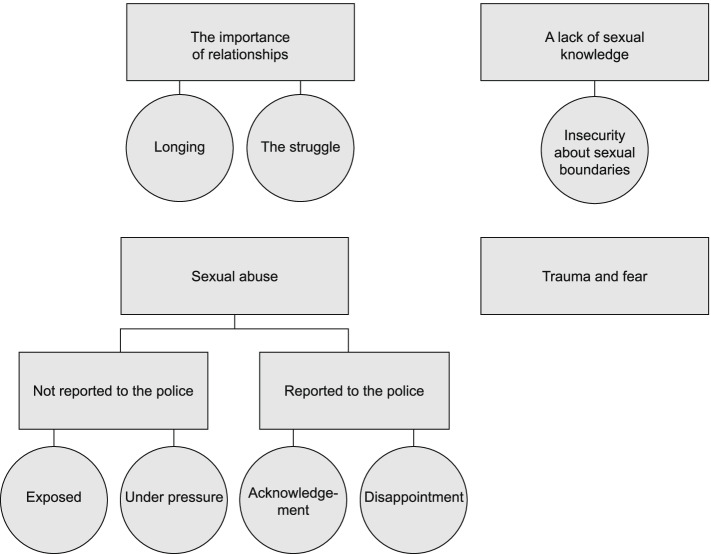
An overview of the results section

Data were analysed using thematic analysis, in line with Braun and Clarke [39]. The first author transcribed the interviews. All authors read the transcripts as a first step, and overall impressions were reviewed jointly. During the second step, the first and second authors individually searched through the transcripts for themes, and then discussed these initial themes until an agreement was reached. A thematic map was created. During phase three, all authors reviewed the thematic map, and some themes were merged. In phase four, a final thematic map was created with four main themes and two subthemes regarding the Sexual abuse theme. In this phase, all authors considered the validity of the candidate themes by proposing and discussing alternative interpretations, and calibrated the data with the candidate themes to check for inconsistencies. During this process, we revised the thematic map another time, to clarify the most important results (See Fig. 1).

## Results

At the start of the interviews, several participants said they appreciated the invitation to the study and highlighted the importance of participating. Some said they had never talked about sexuality with anyone. Four main themes were identified: 1) “The importance of relationships”, 2) “A lack of sexual knowledge”, 3) “Sexual abuse”, a) not reported to the police, b) reported to the police, and 4) “Trauma and fear”.

### The importance of relationships

A central theme in the material concerns being in a relationship and what the participants do to establish or maintain romantic relationships.

#### Longing

Several participants said that they want a relationship and highlight its importance. Many said that being connected to someone/a significant other is more important than having sexual relations. One participant (no. 3) said: “Sex is not important.” Another participant (no. 6) said she has no expectations of sex, and “It is not good to ([have sex] too often either, stick together as much as possible as a team”. One participant (no. 1) said that sex is essential: “For me, it is essential, um, because then you express love for each other.” One participant (no. 7) said: “I want a relationship and a real family, and my dream is to have someone very stable and who respects me as I am.” She continued: “Who can take responsibility and accept my family and me (…) a serious and stable relationship.” Another participant (no. 2), who has previously been abused, said: “I miss having a boyfriend a bit, going out or talking together and things like that, doing nice things outside. Someone I can trust. From what I have experienced, I have been thinking about it, but I’m still a little bit afraid.”

#### The struggle

One participant (no. 5) said that she wants a boyfriend: “But I find it difficult.” She said: “Right now, I’m paying for a monthly membership on Tinder, but I’m not getting any likes. I won’t get any matches anyways. Or I will get a match, but they don’t contact me; I try to contact them but then I [the match] will be deleted.” She says: “Sometimes I feel that my disability is very much in the way.” A little later in the interview, she said: “Yes, in general, it [relationships] is more difficult for us with disabilities.”

Another participant (no. 4) was actively searching for a partner online. He said that he has met several people online, and, on one occasion, the unknown boyfriend of a woman he planned to meet, not the woman herself. He said: "I often end up meeting people that have some experience and that exploit men in a way." He noted that many people want to have a one-night stand, not a relationship. Therefore, he was trying a new tactic involving chatting with people online for a long time to get to know them well. At the time of the interview he was in contact with three different people online.

### A lack of sexual knowledge

Many of the participants stated that they were either in a relationship or had been in the past, and all said that they had had at least one sexual experience. At the same time, several did not understand specific concepts of basic sexual knowledge, such as what sexual orientation is. Moreover, in terms of appropriate ages for potential partners, and some believe, for instance, that a couple of 28 and 19 years or one of 40 and 21 years has a good age range. One participant had heard that “age is just a number” about her relationship with a partner who is 23 years her senior.

#### Insecurity about sexual boundaries

When consent to sex was discussed, several participants could not accurately explain it. Regarding sexual acts, one participant (no. 5) explained that sexual consent means signing a paper. Another participant (no. 3) compared sexual consent to working as a volunteer and sexual consent means that you have given your approval. These statements are not necessarily wrong, but do not touch the essence of sexual consent. After being presented with vignettes about situations of unclear sexual consent, several participants changed their minds about what they thought was acceptable. A participant (no. 3) was presented with a vignette asking if it is okay for a person to change their mind about sex when they are kissing someone, they answered: “Yes, I guess, maybe,” and “It probably is.” Later in the interview, he became more certain and answered clearer of what one is and is not allowed to do. Another participant (no. 1) revised her earlier answer that something was “fine”, saying that she no longer thought so.

One participant (no. 4) said that he got a strange gut feeling before having sex with a woman, so he withdrew from the situation. He elaborated: “Sexual consent is important for the woman, but not for my part. I do it for the woman’s sake, of course, I say ‘yes’.” From this quote, it can be understood that he does not feel that sexual consent applies to him, but is only relevant for the woman.

### Sexual abuse not reported to the police

Several participants talked about experiences of sexual abuse which had not been reported to the criminal justice system.

#### Exposed

One participant (no. 5) shared their thoughts about people with more severe intellectual disabilities who may be exposed to sexual abuse and who are unable to act or defend themselves: “I think a lot when it comes to this kind of situations and those who cannot speak for themselves, people that are a little more disabled than I am.”

Participants also had experiences of unreported sexual abuse. During the interview, a participant (no. 1) said that she had never been abused, but after talking for a while, she remembered being abused when she was 13 years old. She had been waiting for a family member outside her school when a random man came and grabbed her breast which terrified her. Fortunately, the family member was close by and saw what happened; they started to run towards her and the man ran away.

#### Under pressure

Several participants said they have felt pressured to send nude photos to boyfriends/girlfriends or others who ask. However, they regretted it afterwards, and were afraid that their pictures will be shared further. Participant (no. 6) mentioned that the police can help take down a picture that has been posted online. One participant (no. 1) said: “I have been afraid when a man has threatened to post pictures of me online (…), And it’s my fault, I should never have sent pictures to people I don’t know.” She also said her husband has threatened to post pictures of her online: “Yes, it was when he got mad at me, but I knew it would never happen.”

Another participant (no. 5) talked about what they perceive as pressure from a friend: “I feel the pressure, he is constantly asking, and, for some reason, I don’t dare to say no, and I do it. I say sorry (after sending the picture), but I delete you [the match]. I cannot take it anymore.” She continued: “You are being pressured. ‘Why don’t you want to send it? Why?’”.

### Sexual abuse reported to the police

Most of the participants stated that they have been abused by someone they were in a relationship with, by people in positions of power, or by complete strangers. As many as four out of seven of the participants reported contact with the police for this reason.

#### Acknowledgement

One participant (no. 2) said that a person in a higher position at work had sexually abused her: “He began to flirt with me at work and give me attention. I rarely get attention from men.” He also came to her house, and she said: “I think it was disgusting, old men you see on television. He called me a whore, so not particularly nice. He was a little harsh.” She continued: “It took a while before I told the police, I got scared, but I got help from my family and pointed out that the man was incarcerated.

Another participant (no. 5) spoke about experiencing abuse as a customer. She described: “I was about to be raped, but I managed to stop it in time.” A man had started to touch her, but she managed to stop it before he got further down on her body, and she got away. She said that she asked him to write down his phone number on her receipt and got away by pretending she would contact him later: “Smart for me, but stupid for him. I am a good talker, and I said: I’ll call you when I need more ‘help’ or something. The day after, I got a hold of (…) and (…) [named staff], and I got straight to the police station and reported him, giving him [a police officer] the receipt with the phone number.”

One participant (no. 4) experienced being reported for a sexual assault, but the police investigation revealed that their accuser had lied. Another participant (no. 7) told of experiencing assault. She said she pushed the assailant away and explained that she had defended herself in the same way during a previous sexual assault. She described many assaults, randomly on the street and outside her home. She talked about threats from one perpetrator: “Do this. If you don’t, you will not get the call phone.” She also talked about an instance where a male staff member tickled her friend. She wanted to defend her friend, but the man walked towards her and touched her breast and she froze. The participant said that, during another assault, she managed to contact the police:

*And I managed to report him, too. They (the police) took DNA traces from me and the criminal. He was punished and put in prison for almost five or six months. He also had to compensate me with nearly 150 000 NOK.*

She described meetings and facilitated interviews with a lawyer and the police. She had received instructions about DNA from the police, and she demonstrated good knowledge of DNA and the importance of gathering it.

#### Disappointment

One participant (no. 7) said that she and a friend had reported a person for sexual abuse, but that both of their cases were dropped. She was asked how she felt when the case was dropped, and she said: “It’s almost like banging your head against the wall. Nothing is happening, and it is sad. It is very uncomfortable afterwards when the person who has been disgusting doesn’t face any consequences.”

### Trauma and fear

Some participants reported fear and trauma after being sexually abused. They described knowledge that they did not have at the time of the sexual abuse, but which they acquired afterwards.

One participant (no. 2) reported that she was anxious and tense after experiencing sexual abuse. She said: “I received compensation, but it doesn’t help much. I have a lot of trauma in my head. It doesn`t go away.” Another participant (no. 7) talked about her own physical experience during the assault. She said:

*I was speechless. My whole body was completely frozen. Shock. It was very uncomfortable. I did not quite know what to do, but I saw he had put the cell phone on the grass. I looked at the clock. Time passed. As time passed by, my brain had to think. One of my brains said, “Take it.” The other brain said, “Get away.”*

One participant (no. 7), who had been exposed to sexual abuse several times, described incidents where she had been offered money from a stranger one late evening downtown. Another random man tried to touch her. From experiencing an earlier assault, she had learned to defend herself by saying: “Leave me alone. I do not want anything from you. You are a stranger to me.” She continued: “It is because I obviously have had a lot of negative experiences that have made me sceptical. I have learned to defend myself in a way.”

Some participants explained that they have been scared, and refer to guilt related to the experienced sexual abuse. One participant (no. 2) said:

*Yes, I was scared, and it was a little bit my fault. I did not want to say it. I was afraid and did not dare to tell anyone, but I have heard them say that it was not my fault.*

From this quote, it seems that she is not convinced that she is entirely innocent, even though others have told her it is not her fault. Another participant (no. 7) said that she was sexually abused in exchange for something she wanted. She said:

*He is a stranger, and you should stay far away from strangers. But I didn’t know that. I had no idea. I was mainly interested in the cell phone he had, and I did not understand what he was thinking. It’s like what you call an afterthought—something I did not understand, not at all, but what I felt wasn’t good, and I had to get away* [speaking quietly and slowly].

The same participant also talked about an episode where she brought a stranger home: *Actually, I should not invite a stranger home to my place, but I did not know because I was only 18 years old. I had no clue about that.*

## Discussion

In the present study, we found that the participants wanted romantic relationships, and that for some, sex was of lesser importance. Many participants have had trouble finding someone to have a romantic relationship with. The participants lacked adequate knowledge about sexual consent though many could explain the concept of consent in other contexts. Many participants reported that they had experienced sexual abuse in real life and online. Most participants who reported the sexual abuse had positive experiences with assistance from the police and the criminal justice system. The participants experiencing sexual abuse reported of trauma and fear related to their experiences.

The importance of being connected to someone/a significant other and being in an intimate relationship was fundamental to the participants. These results align with previous studies [10, 15]. Some of the participants reflected on their difficulties finding someone to have a romantic relationship with, and the risks they exposed themselves to in their search. This results may explain why so many people with intellectual disabilities have sex they do not want to have [25]. This study reveals that the participants lacked essential knowledge related to sexual consent, which we suggest is crucial in decreasing the risk of sexual abuse. Further, when people with intellectual disabilities are not clear about the meaning of the term sexual consent, it can complicate a police investigation. An explanation of whether there was sexual consent can be decisive in whether an incident is considered to be a criminal act or not. The results of this study show that the participants are insecure about sexual consent, and some felt pressured to send nude pictures. Several studies have investigated sexual education for people with intellectual disabilities. McDaniels and Fleming [40] found that formal, personalised and precise sexual education is lacking. A literature review [4] has identified a need for improved education and support for people with intellectual disabilities to access information concerning sexual health. Schaafsma, Kok [41] state it would be beneficial that sexual education target sexuality-related skills. Some people with intellectual disabilities have limited knowledge about sexual consent and awareness of one’s own sexual rights. We believe that practical education, teaching in explicit terms and expanding vocabulary for discussing feelings, sexual relations and personal preferences can help people with intellectual disabilities navigate the sexual landscape and decrease the risk of exploitation and sexual abuse. Equally important as sexual education are environmental factors so that people with intellectual disabilities can experience a healthy sexual relationship. A broader perspective has been reported by Matin, Ballan [42]. In addition to a lack of sexual education, they also found that women with intellectual disabilities reported barriers, such as the lack of support and being controlled by their surroundings, such as families or institutions. The present study confirms findings from previous research and should encourage policymakers to strengthen education for future practitioners in schools and institutions. Hence, to meet the sexual health needs of people with intellectual disabilities.

Several of the participants spoke openly about their experiences of sexual abuse and shared thoughts about their increased risk of sexual abuse because of their disabilities. Our results align with those of previous studies [23], which found that people with intellectual disabilities are more vulnerable to victimisation. Further, the participants have experienced online sexual abuse; Gil-Llario, Diaz-Rodriguez [43] point out that the prevention of online victimisation has recently become especially urgent as during the pandemic more people with intellectual disabilities expressed their sexuality online.

One unexpected finding was that several participants had had positive experiences obtaining assistance from the police and the criminal justice system. These results are contrary to Murphy-Oikonen, McQueen [44], who found that women with vulnerabilities experienced not being believed by the police when reporting sexual abuse. The participants in our study are service users of SHCS, making it more likely that they were identified by the criminal justice system as people with intellectual disabilities, and treated accordingly. Thus, when a diagnosis of intellectual disability is known to the criminal justice system, it is more likely that the police-investigation will be adjusted appropriately. It is also possible that recent progress based on the UN convention (CRPD) on awareness of intellectual disabilities among the police, including facilities like the Barnahus, affected our participants experiences.

It is striking how respondent no. 1 altered her perspective during the interview and recognized that she had been the victim of exploitation. She had not considered her experiences in this way before. Her former viewpoint may be explained by the criminal justice system giving more attention to offenders than victims.

Emotional distress described by the responders was often a consequence of exploitation and insufficient knowledge of sexuality and consent. Being treated respectfully by the police or receiving compensation for victimisation did not make up for the participants’ frightening experiences. These findings support previous research [45] highlighting the need for women with intellectual disabilities to be informed about sexually abusive relationships and ways to get help. In our study, one male participant (no. 4) identified himself as vulnerable and felt that he had been exploited by women for sex. Therefore, we want to raise awareness of the importance of providing men with intellectual disabilities with this same information. Efforts to implement preventive measures should include targeted education about exploitation in friendly, romantic and sexual relationships. Moreover, our findings agree with Levine, Proulx [46], who emphasised the need to increase trauma treatment for people with intellectual disabilities.

### Limitations

This study aimed to explore perceptions and knowledge of adults with intellectual disabilities regarding personal and sexual boundaries. We acknowledge that there are some limitations. The participants were recruited as a convenience sample at an SHCS service. We recruited people with verbal abilities who were able to engage in conversation on (share their knowledge about) the subject. We included only seven participants in the present study, and male participants were underrepresented. The selection of participants was based on the ability to participate in the interviews, which excluded people lacking sufficient communication skills. A limitation of the study is some of the questions being asked. For some, questions about sexuality and former sexual education are complex and require the ability to remember and generalise. The first author tried to meet the participants on equal terms, and we think it is essential to ask these questions in a study like this. Some participants provided more information than others, which is expected in qualitative studies. We recruited as many participants as possible within the project’s time frame to include multiple experiences. An unexpected outcome from this study are the insights about the follow-up from the police, and how the participants experienced moreover positive relations. The knowledge obtained on this matter is vital, however more interview data is needed to explore this comprehensively. The self-presentations of participants in this study may be affected by a tendency to answer questions in the way of social desirability that will be viewed favourably by others according to prevailing standards of behaviour and thought [47]. Moreover, the results of the present study add to the existing research. Despite its exploratory nature, this study offers some valuable insights that can contribute to our understanding of the blurred distinction between personal and sexual relationships which may, in turn, help to prevent sexual abuse.

## Conclusion

This study has shown that people with intellectual disabilities long for romantic relationships but have trouble finding safe and reliable partners. The participants are insecure about their knowledge of consent relating to sexual activity, and several reported experiencing sexual abuse, including online. Notably, the participants who had reported the sexual abuse had positive experiences obtaining assistance from the police and the criminal justice system. Some participants reported trauma and fear related to the experienced sexual abuse. This study provides several important insights which require further research. Our findings highlight the need for more research about the follow-up from the police after people with intellectual disabilities experience sexual abuse. Future studies should address experiences of trauma in people with intellectual disabilities who have been sexually abused.

## Data Availability

The dataset generated and/or analysed during this study is not publicly available due to the sensitive and personal information contained. Data may be available from the corresponding author on reasonable request and following ethical approval.

## References

[1] World Health Organization (2006). Defining sexual health: Report of a technical consultation on sexual health, 28–31, January 2002, Geneva.

[2] Azzopardi-Lane C, Callus A (2015). Constructing sexual identities: People with intellectual disability talking about sexuality. Br J Learn Disabil.

[3] World Health Organization. Sexual health 2022. Available from: https://www.who.int/health-topics/sexual-health#tab=tab_1. [Cited 2022 08.08].

[4] Borawska-Charko M, Rohleder P, Finlay WML (2016). The sexual health knowledge of people with intellectual disabilities: a review. Sex Res Social Policy.

[5] Jahoda A, Pownall J (2014). Sexual understanding, sources of information and social networks; the reports of young people with intellectual disabilities and their non-disabled peers: sexual understanding and young people. J Intellect Disabil Res.

[6] McCabe MP, Cummins RA, Deeks AA (1999). Construction and psychometric properties of sexuality scales: sex knowledge, experience, and needs scales for people with intellectual disabilities (SexKen-ID), people with physical disabilities (SexKen-PD), and the general population (SexKen-GP). Res Dev Disabil.

[7] Murphy GH, O'Callaghan ALI (2004). Capacity of adults with intellectual disabilities to consent to sexual relationships. Psychol Med.

[8] O'Callaghan AC, Murphy GH (2007). Sexual relationships in adults with intellectual disabilities: understanding the law. J Intellect Disabil Res.

[9] Eastgate G, Scheermeyer E, van Driel ML, Lennox N (2012). Intellectual disability, sexuality and sexual abuse prevention: a study of family members and support workers. Aust Fam Physician.

[10] Lafferty A, McConkey R, Taggart L (2013). Beyond friendship: the nature and meaning of close personal relationships as perceived by people with learning disabilities. Disabil Soc.

[11] Healy E, McGuire BE, Evans DS, Carley SN (2009). Sexuality and personal relationships for people with an intellectual disability. Part I: service-user perspectives. J Intellect Disabil Res.

[12] Rojas S, Haya I, Lázaro-Visa S (2016). 'My great hope in life is to have a house, a family and a daughter': relationships and sexuality in intellectually disabled people. Br J Learn Disabil.

[13] Lesseliers J, Van Hove G (2002). Barriers to the development of intimate relationships and the expression of sexuality among people with developmental disabilities: their perceptions. Res Pract Persons Severe Disabil.

[14] Eastgate G, van Driel ML, Lennox N, Scheermeyer E (2011). Women with intellectual disabilities: a study of sexuality, sexual abuse and protection skills. Aust Fam Physician.

[15] Sullivan F, Bowden K, McKenzie K, Quayle E (2013). ‘Touching people in relationships’: a qualitative study of close relationships for people with an intellectual disability. J Clin Nurs.

[16] Nixon M, Thomas SDM, Daffern M, Ogloff JRP (2017). Estimating the risk of crime and victimisation in people with intellectual disability: a data-linkage study. Soc Psychiatry Psychiatr Epidemiol.

[17] Brown M, McCann E (2018). Sexuality issues and the voices of adults with intellectual disabilities: a systematic review of the literature. Res Dev Disabil.

[18] Dukes E, McGuire BE (2009). Enhancing capacity to make sexuality-related decisions in people with an intellectual disability. J Intellect Disabil Res.

[19] Kucuk S, Platin N, Erdem E (2017). Increasing awareness of protection from sexual abuse in children with mild intellectual disabilities: an education study. Appl Nurs Res.

[20] Hickson L, Khemka I, Golden H, Chatzistyli A (2015). Randomized controlled trial to evaluate an abuse prevention curriculum for women and men with intellectual and developmental disabilities. Am J Intellect Dev Disabil.

[21] United Nations (2006). Article 25 – Health. Convention on the rights of persons with disabilities.

[22] Thompson VR, Stancliffe RJ, Broom A, Wilson NJ (2014). Barriers to sexual health provision for people with intellectual disability: a disability service provider and clinician perspective. J Intellect Dev Disabil.

[23] Codina M, Pereda N, Guilera G (2020). Lifetime victimization and poly-victimization in a sample of adults with intellectual disabilities. J Interpers Violence.

[24] Tomsa R, Gutu S, Cojocaru D, Gutierrez-Bermejo B, Flores N, Jenaro C (2021). Prevalence of sexual abuse in adults with intellectual disability: systematic review and meta-analysis. Int J Environ Res Public Health.

[25] Brkić-Jovanović N, Runjo V, Tamaš D, Slavković S, Milankov V (2021). Persons with intellectual disability: Sexual behaviour, knowledge and assertiveness. Zdravstveno Varstvo.

[26] Straffeloven. Lov om straf kapittel 14 § 77. Skjerpende omstendigheter [Aggravating circumstances]:. 2020.

[27] American Association on Intellectual and Developmental Disabilities. Defining Criteria for Intellectual Disability 2022. Available from: https://www.aaidd.org/intellectual-disability/definition. [Cited 2022 11.05].

[28] Henshaw M, Thomas S (2012). Police encounters with people with intellectual disability: prevalence, characteristics and challenges: police encounters with people with intellectual disability. J Intellect Disabil Res.

[29] Antaki C, Richardson E, Stokoe E, Willott S (2015). Can people with intellectual disability resist implications of fault when police question their allegations of sexual assault and rape?. Intellect Dev Disabil.

[30] Beckene T, Forrester-Jones R, Murphy GH (2020). Experiences of going to court: witnesses with intellectual disabilities and their carers speak up. J Appl Res Intellect Disabil.

[31] Statens Barnehus. The childrens house Oslo 2022. Available from: https://www.statensbarnehus.no/barnehus/statens-barnehus-oslo/information-in-english/.

[32] Vik BF, Nöttestad JA, Schei B, Rasmussen K, Hagemann CT (2019). Psychosocial Vulnerability among patients contacting a Norwegian Sexual Assault Center. J Interpers Violence.

[33] Vik BF, Rasmussen K, Schei B, Hagemann CT (2020). Is police investigation of rape biased by characteristics of victims?. Forensic Sci Int Synerg.

[34] Åker TH, Johnson MS (2020). Sexual abuse and violence against people with intellectual disability and physical impairments: Characteristics of police-investigated cases in a Norwegian national sample. J Appl Res Intellect Disabil.

[35] Søndenaa E, Olsen T, Kermit PS, Dahl NC, Envik R (2019). Intellectual disabilities and offending behaviour: the awareness and concerns of the police, district attorneys and judges. J Intellect Disabil Offending Behav.

[36] Braun V, Clarke V (2013). Successful qualitative research: a practical guide for beginners.

[37] Helsedirektoratet. 2.8. Vansker knyttet til seksualitet i målgruppen [2.8 Difficulties related to sexuality in the target group] 2015. Available from: https://www.helsedirektoratet.no/veiledere/prioriteringsveiledere/habilitering-av-voksne-i-spesialisthelsetjenesten/tilstander-for-habilitering-av-voksne-i-spesialisthelsetjenesten/vansker-knyttet-til-seksualitet-i-malgruppen#vansker-knyttet-til-seksualitet-i-malgruppen-alvorlige-veiledende-frist-4-uker. [Updated 06.10.22; Cited 2022 10.04.22].

[38] Ministry of Justice and Public Security. The penal code. Section 196. Duty to avert a criminal offence. 2021.

[39] Braun V, Clarke V (2006). Using thematic analysis in psychology. Qual Res Psychol.

[40] McDaniels B, Fleming A (2016). Sexuality education and intellectual disability: time to address the challenge. Sex Disabil.

[41] Schaafsma D, Kok G, Stoffelen JMT, Curfs LMG (2017). People with intellectual disabilities talk about sexuality: implications for the development of sex education. Sex Disabil.

[42] Matin BK, Ballan M, Darabi F, Karyani AK, Soofi M, Soltani S (2021). Sexual health concerns in women with intellectual disabilities: a systematic review in qualitative studies. BMC Public Health.

[43] Gil-Llario, Díaz-Rodríguez I, Morell-Mengual V, Gil-Juliá B, Ballester-Arnal R. Sexual Health in Spanish People with Intellectual Disability: the Impact of the Lockdown due to COVID-19. Sex Res Soc Policy. 2021;19(3):1217–27. 10.1007/s13178-021-00621-7. 10.1007/s13178-021-00621-7 PMC8308064 34335991

[44] Murphy-Oikonen, McQueen K, Miller A, Chambers L, Hiebert A. Unfounded Sexual Assault: Women’s Experiences of Not Being Believed by the Police. J Interpersonal Violence. 2022;37(11-12):NP8916–NP8940. 10.1177/0886260520978190. 10.1177/0886260520978190 PMC9136376 33305675

[45] McCarthy M, Bates C, Triantafyllopoulou P, Hunt S, Milne SK (2019). “Put bluntly, they are targeted by the worst creeps society has to offer”: Police and professionals' views and actions relating to domestic violence and women with intellectual disabilities. J Appl Res Intellect Disabil.

[46] Levine KA, Proulx J, Schwartz K (2018). Disconnected lives: Women with intellectual disabilities in conflict with the law. J Appl Res Intellect Disabil.

[47] Kittelsaa AM (2014). Self-presentations and intellectual disability. Scand J Disabil Res.

